# Rational Design of Temperature-Sensitive Alleles Using Computational Structure Prediction

**DOI:** 10.1371/journal.pone.0023947

**Published:** 2011-09-02

**Authors:** Christopher S. Poultney, Glenn L. Butterfoss, Michelle R. Gutwein, Kevin Drew, David Gresham, Kristin C. Gunsalus, Dennis E. Shasha, Richard Bonneau

**Affiliations:** 1 Department of Biology, Center for Genomics and Systems Biology, New York University, New York, New York, United States of America; 2 Computer Science Department, Courant Institute of Mathematical Sciences, New York University, New York, New York, United States of America; University of South Florida College of Medicine, United States of America

## Abstract

Temperature-sensitive (ts) mutations are mutations that exhibit a mutant phenotype at high or low temperatures and a wild-type phenotype at normal temperature. Temperature-sensitive mutants are valuable tools for geneticists, particularly in the study of essential genes. However, finding ts mutations typically relies on generating and screening many thousands of mutations, which is an expensive and labor-intensive process. Here we describe an *in silico* method that uses Rosetta and machine learning techniques to predict a highly accurate “top 5” list of ts mutations given the structure of a protein of interest. Rosetta is a protein structure prediction and design code, used here to model and score how proteins accommodate point mutations with side-chain and backbone movements. We show that integrating Rosetta relax-derived features with sequence-based features results in accurate temperature-sensitive mutation predictions.

## Introduction

The study of essential genes – those genes that result in inviability of the organism or cell when nonfunctional – poses a unique challenge to the *in vivo* study of gene function. In model organisms such as *D. melanogaster*, *C. elegans* and *S. cerevisiae* the use of conditionally inactivated alleles has proved a fruitful method for the study of essential gene function within the context of the organism. The ability to control the inactivation of an essential gene enables studies of the consequence of functional inactivation of essential genes and the identification of genetic interactions by means of genetic suppression studies. These studies are frequently informative of the pathways and complexes in which the gene product participates. A critical requirement is that a functional and nonfunctional state is possible for the same allele and that these states can be experimentally controlled. Although a variety of methods exist for the regulated inactivation of essential genes including the use of gene expression induction/repression systems, the workhorse of essential gene studies has long been temperature sensitive (ts) alleles. Typically, these alleles produce a functional gene product at one temperature (the permissive temperature) but are rendered non-functional at a higher – or occasionally lower – temperature (the restrictive temperature).

The main challenge in the use of ts mutations is the difficulty of discovering or generating them. Methods for generating ts mutations fall into three general categories: random methods, procedure-based methods, and predictive methods. Random methods, such as mutation with ethyl methanesulfonate or PCR mutagenesis, make many random mutations to the genome or to a specific gene of an organism. Random mutation is necessarily followed by extensive screening to isolate the small number of resulting ts mutations, if any. Procedure-based methods rely on specific techniques, such as the fusion of a temperature-sensitive N-degron [1] to a protein, that induce a ts phenotype. We also place “naïve” non-random techniques such as alanine scanning [2] in this group. Procedure-based methods remove the need for extensive screening imposed by random methods, but are limited in other ways: N-degron fusion and similar techniques provide no recourse should that specific technique fail, and may also introduce side effects by making a larger-scale perturbation to the protein instead of a simple amino acid substitution, while alanine scanning generally produces surface mutations that tend to disrupt particular interactions rather than overall protein function.

Accurate predictive techniques promise the advantages of both random and procedure-based techniques. By predicting a small number of high-likelihood substitutions, they avoid the need for screening thousands of mutants. The mutations are produced by making straightforward single-amino acid substitutions, and if the initial predictions fail to produce a ts phenotype, one can move farther down the ranked list of predictions, though obviously at the cost of more screening. Previous work in predicting temperature-sensitive mutations has been limited to prediction based on sequence [3]–[5]. These techniques focus on predicting amino acid burial (where buried amino acids are those below a threshold for solvent-accessible surface area) from sequence since mutations to buried amino acids are more likely to produce a ts phenotype. Once putative buried amino acids have been identified, substitutions are made at these sites and the resulting mutants are screened for a ts phenotype. Here we present a method that explicitly uses protein structure in the prediction process.

Predicting temperature-sensitive mutations presents a number of challenges. The first is that a ts phenotype may manifest itself in many ways. A ts protein may exhibit a reduction in stability or solubility; it may acquire resistance to proteolysis, or be cleared more quickly because of partial unfolding; it may show reduced function; or it may not accumulate in sufficient quantity because of poor expression, failure to fold, or aggregation. From this list of potential causes, we chose to focus on reduced stability, because reducing stability affects a protein's function generally instead of interfering with single interactions. In addition, reduced stability is more tractable for computational modeling: a reduction in stability will be reflected as a lowering of 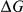, the free energy of unfolding of the protein, which will be reflected in the energy function of protein modeling software such as Rosetta [6], [7]. We restrict our search to buried sites because mutations at buried sites a) are correlated with both reduced stability and a ts phenotype [8], and b) are more likely to perturb the entire function of a protein rather than a single interaction. For proteins of known function a more directed design approach could be adopted, but we choose to focus on a method of general utility in the study of poorly understood proteins.

Quantifying the effects of mutations on protein structure presents its own challenges. Proteins vary tremendously in structure and function, variations that are reflected in their wide range of native stabilities. While small reductions in stability (a decrease in 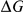 or a negative 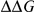) may be tolerated and large changes will most likely result in a loss of function phenotype, ts mutations occupy a middle ground between toleration and loss of function. Although changes in the score calculated by Rosetta's energy function due to single amino acid substitutions have been shown to correlate well with experimentally measured 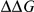 values [9], 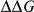 alone is not sufficient to predict whether a mutation will result in a ts phenotype. Therefore, instead of directly comparing energies of native and mutated structures, we accommodate the natural variation in structure and function by 1) generating a distribution of component Rosetta score terms (Table 1) across multiple candidate structures for each mutation, and 2) comparing mutant structure and native structure distributions rather than comparing energy function terms directly. We then use these distribution comparisons as inputs to a machine learning algorithm, allowing us to pinpoint the intermediate range of destabilization that is most likely to yield a ts phenotype.

**Table 1:** Rosetta score terms and derived features.

Feature	Description
score	overall score: weighted sum of other score terms
fa_atr^1^	Lennard-Jones attractive component
fa_rep	Lennard-Jones repulsive component
fa_sol^1^	Lazaridis-Karplus solvation energy
fa_intra_rep^1^	LJ repulsive between same-residue atoms
pro_close	proline ring closure energy
fa_pair	pair term, statistics-based: electrostatics, disulfides
hbond_sr_bb	H-bonds: backbone-to-backbone, close in sequence
hbond_lr_bb	H-bonds: backbone-to-backbone, distant in sequence
hbond_bb_sc	H-bonds: backbone-to-side chain
hbond_sc	H-bonds: side chain-to-side chain
dslf_ss_dst	disulfide bond S-S distance score
dslf_cs_ang	disulfide bond C 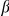 -S-S angles score
dslf_ss_dih	disulfide bond S-S dihedral score
dslf_ca_dih	disulfide bond C 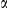 -C 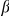 -S-S dihedrals score
rama	probability of 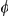 , 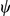 angles given amino acid identity and secondary structure
omega	deviation of 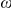 bond dihedral angle from ideal of 180 degrees
fa_dun	rotamer self-energy from Dunbrack library
p_aa_pp	probability of amino acid given 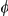 , 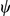
ref	reference state (unfolded) energy
Repack_average_score	average of overall score across 3 relax iterations
Repack_stdev_score	stdev of overall score across 3 relax iterations
gdtmm1_1	maxsub fraction: maxsub term/# residues, using maxsub rms thresh = 1.0 and distance thresh = 1.0
gdtmm2_2	maxsub fraction: rms thresh = 2.0, distance thresh = 2.0
gdtmm3_3	maxsub fraction: rms thresh = 3.0, distance thresh = 3.0
gdtmm4_3	maxsub fraction: rms thresh = 4.0, distance thresh = 3.0
gdtmm7_4	maxsub fraction: rms thresh = 7.0, distance thresh = 4.0
irms^2^	RMS from input structure
maxsub^1^	size of C 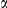 atom subset that a) can be aligned to native within rms threshold = 4.0, and b) are within distance threshold = 7.0 [24]
maxsub2.0	maxsub w/rms thresh = 2.0 and distance thresh = 3.5
rms^1^	RMS from native

1 Removed due to high correlation with other feature(s).

2 Always zero.

Rosetta score terms and descriptions. Three features were derived from each Rosetta score term, denoted by suffix Q1, Q2, or Q3, based on mutant distribution quartiles 1–3 as described in Methods and Fig. 5. Superscripts denote feature groups removed from the final training set.

## Methods

**Figure 1:**
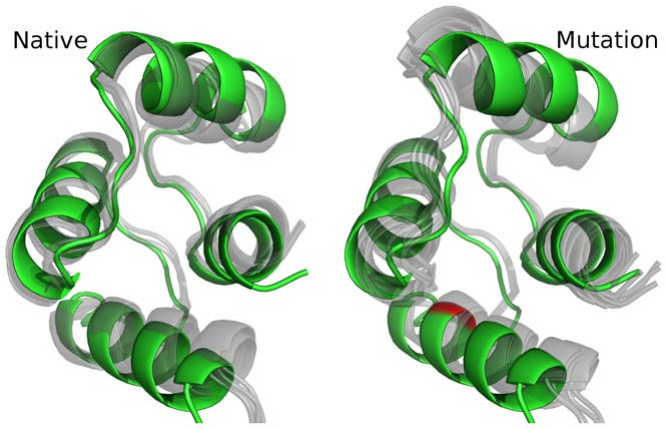
Typical ensembles of structures produced by Rosetta relax runs for calmodulin. Shown here are structures generated by Rosetta relax runs that allow protein structures to “relax” to a lower energy state. The starting structure – one domain of yeast calmodulin – is shown in green, and the generated structures are shown in gray, with runs starting from the native structure on the left and runs from a mutation (F89I) on the right. The mutated site is shown in red in the mutant structure. The wt ensemble shows less variation in both difference from the starting structure and difference within the ensemble than the mutation ensemble. The differences between wild-type and mutation ensembles are quantified by comparing distributions of Rosetta score terms.

**Figure 2:**
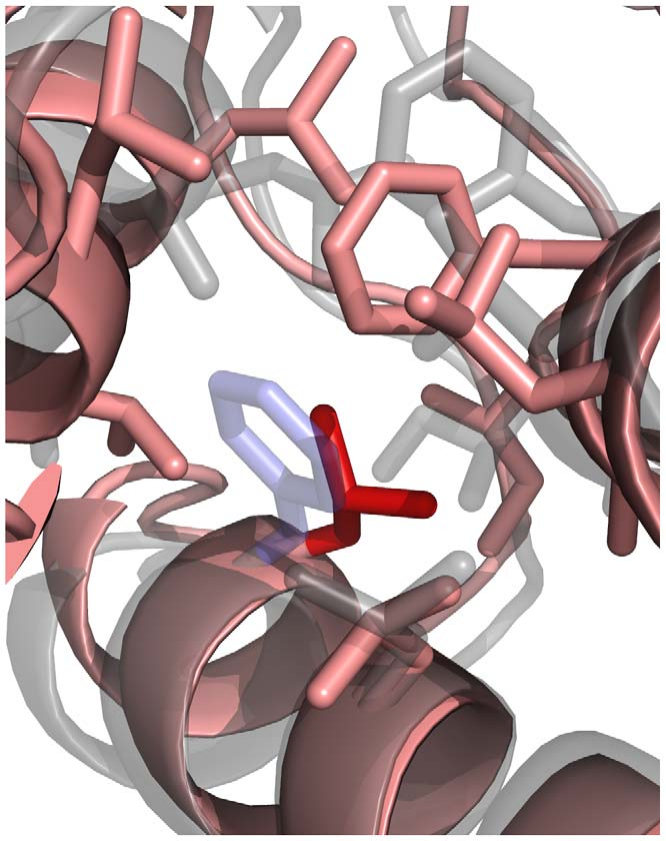
Effects of a single amino acid mutation. Shown here is a Rosetta-generated structure for one mutation (F89I) to yeast calmodulin. The relaxed starting structure is shown in transparent gray, the mutant structure in pink, the native phenylalanine at position 89 in transparent blue, and the mutation to isoleucine in solid red. The mutated structure has accommodated the F89I mutation by small backbone movements, such as the shift in helix position at residue 89, and reconfiguration of nearby side chains.

**Figure 3:**
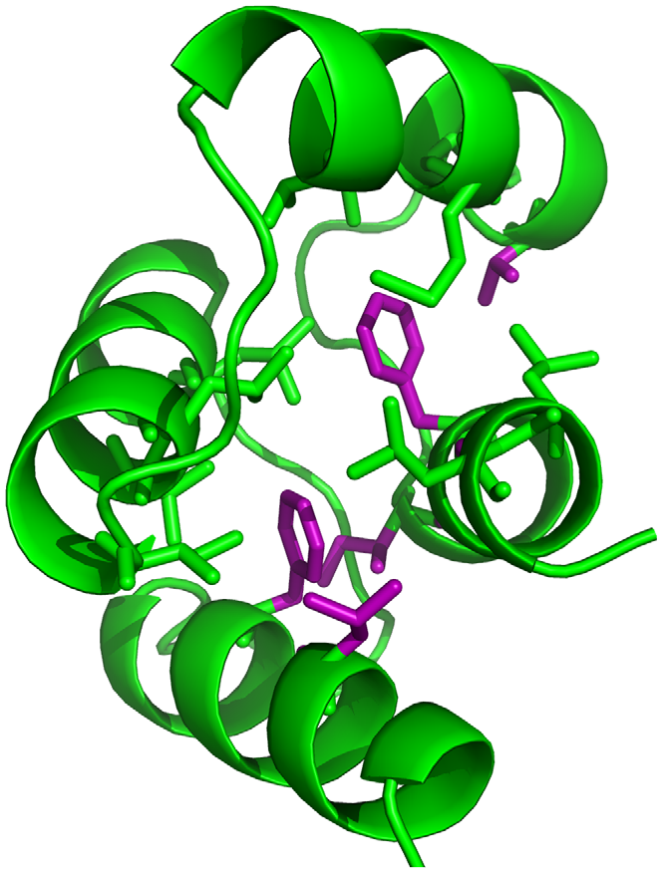
Sites of predicted temperature-sensitive mutations. The crystal structure of one domain of yeast calmodulin is shown in cartoon representation in green. Residues in the hydrophobic core are shown as green sticks, and hydrophobic core residues with predicted ts mutations are shown in purple. Of the top 20 predictions on calmodulin, 10 each from SVM-LIN and SVM-RBF, 15 mutations occur at these six sites.

Our method takes a protein structure and produces as output a list of proposed amino acid substitutions, ranked by their predicted probability of producing a ts phenotype. The “top 5” list of predicted ts mutations is simply the five highest-ranking mutations from the list of predictions. Our prediction pipeline is as follows:

1. Start with the known structure of a protein of interest, or a high-quality homology model (as defined below).
2. Find the buried sites in the protein of interest, and create models for mutations to all other amino acids at those sites (where buried sites are defined as sites with less than 10 percent solvent-accessible surface area).
3. For each model generated in step 2, run the Rosetta relax protocol multiple times to simulate accommodation of the mutation by the protein. Run the relax protocol on the starting “wild-typ” (wt) structure as well. This results in an ensemble of putative structures for each mutation and for the wild-type structure (Fig. 1).
4. Compare the Rosetta scores of each mutation ensemble to the scores of the wt ensemble to create a set of features that quantify the effect of each mutation on the protein structure (Fig. 2.) At each position, add features such as solvent accessibility and conservation of the native amino acid.
5. Use the features from step 4 to train a classifier to classify the mutations as temperature-sensitive or non-temperature sensitive using a support vector machine (SVM) [10] trained on known ts and non-ts mutations (Fig. 3). Validate this classifier on a leave-out test set.

### Solvent Accessibility and Stability

Solvent-accessible surface area (usually abbreviated ACC or ASA) refers to the surface area of a molecule that is accessible to a solvent [11]. In our case, accessibility is calculated for each amino acid in a protein, and expressed as the fraction or percent of the side chain that is accessible. We restrict our method to sites that are buried, *i.e.*, those residue positions which are 10% accessible or less in the native structure, because a) mutations at buried sites are correlated with reduced stability (decreased 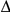
G) and a ts phenotype, and b) surface mutations might be at an interface, and therefore cause a ts phenotype by perturbing a specific interaction of the target protein with another protein.

### Training Set Curation

**Figure 4:**
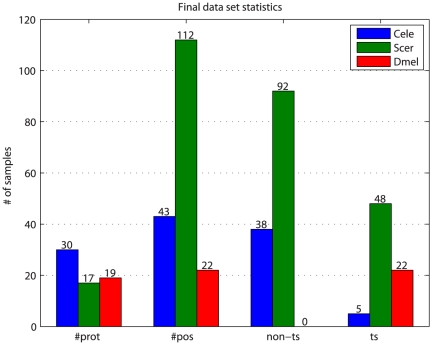
Training set statistics. Counts are shown for the total number of proteins (#prot), positions (#pos), and non-ts and ts samples, separated by species. The training set comprises a total of 205 mutations (75 ts, 130 non-ts) to 177 sites in 66 proteins. Yeast has the largest number of samples, and the most balanced distribution of ts and non-ts samples; worm has only 5 ts samples, and fly lacks non-ts samples. The difference between the number of proteins and the number of positions for yeast is due to the presence of the histone complex data, which comprise many mutations to different positions within the same structure.

We mined available literature to generate a training set of known ts and non-ts examples (or “samples” in the parlance of machine learning). We gathered a set of mutations to worm (*C. elegans*), yeast (*S. cerevisiae*) and fly (*D. melanogaster*) proteins using a combination of database and literature searches. Worm mutants were culled from WormBase [12] v200 and WorTS; yeast mutants were derived from the Saccharomyces Genome Database, Textpresso [13] searches, and the Histone Systematic Mutation Database; and fly mutants were collected from FlyBase [14]. Collecting the training set presented significant challenges: database annotations for a temperature-sensitive phenotype are generally not well standardized, and explicit annotations of “not temperature-sensitive” are essentially non-existent. However, by screening out lower confidence ts examples and using conservative heuristics for finding non-ts examples (*e.g.*, selecting mutations not annotated as temperature-sensitive from papers describing ts mutations), we compiled a set of roughly 1300 ts and non-ts mutations. After selecting for samples that a) had known structures or homology models with at least 70% identity, and b) were at least 90% buried, we arrived at a final training set with 205 samples (75 ts, 130 non-ts) (Fig. 4). This set of mutations has only one pair of homologous proteins (worm and yeast actin), with a single TS site in common between them. Removing this single homologous site does not significantly effect test-set or training-set performance by any metric used in this work.

### Generating Starting Structures/Homology Modeling

The first formal step in the prediction process is to find a structure for the protein of interest. The protocol described here requires an experimental structure or a high quality homology model as described below. We used MODELLER [15] to make homology models for the frequent training set cases for which we had no experimentally determined structure, keeping models with an identity of 70% or better in the domain of the mutation. After determining a starting structure of the domain of interest – either an experimentally established or a homology modeled structure – we found buried sites (10% or less side chain accessibility) using Probe [16]. For a typical protein, 30–50% of its sites will meet this cutoff. At each of these buried sites, we generated *in silico* models for each of 19 possible mutations. This provides a specific advantage over most methods for exploring random mutations by experimental screening, which generate mutations to amino acids whose codons differ by only one nucleotide from the native amino acid.

### Model Relaxation

The models of mutated proteins generated above are not physically accurate without further refinement: they may contain steric clashes or other issues after substitution of one residue for another in the structure. Each mutated model must be allowed to accommodate the mutation before being evaluated as a potential temperature-sensitive mutation. The heart of our method is the use of Rosetta to simulate accommodation of mutations by allowing small backbone and side chain moves prior to evaluating results via the Rosetta energy function. Rosetta is a collection of protocols for predicting and manipulating protein structures: there are protocols for *de novo* structure prediction from sequence, protein design, and protein-protein docking, among others. Each of these protocols relies on Rosetta's energy function, which evaluates structures by calculating different component energy terms (see Table 1) that are then combined as a weighted sum into a final overall score. Some of these terms are based on models of physical properties such as van der Waals forces (energy terms fa_atr, fa_rep, and fa_intra_rep) or solvation (energy term fa_sol); others are derived from the statistics of known proteins, such as the 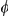 and 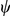 bond angles and amino acid identity at a given site (energy terms rama, p_aa_pp). Our method uses the “fast relax” protocol: given a starting structure, it searches for a lower-energy conformation of the structure, allowing energetically unfavorable features such as steric clashes to be resolved. Fast relax modifies backbone and side chain angles to find low-energy conformations using an optimized form of gradient descent (Rosetta minimization type “dfpmin_armijo_nonmonotone”). During one fast relax protocol run, candidate low-energy conformations are generated during three iterations of the following algorithm: gradient descent is performed six times on the current “best” structure while ramping up the weight of the van der Waals repulsive term. The “best” structure is tracked by a Monte Carlo object that retains lower-energy structures and accepts higher-energy ones according to the Metropolis criterion. The best structure from one iteration of the ramping process becomes the starting structure for the next set, and the lowest-energy structure seen during the entire protocol run is returned as the final result.

The relax protocol does not find a single “best” global low-energy conformation; rather, it reports the lowest-energy conformation seen during the Monte Carlo sampling process, which may or may not represent the actual global minimum. Therefore we perform multiple runs of the fast relax protocol to produce an “ensemble” of 50 low-energy conformations derived from the same starting structure. We then compare the distributions of the score terms of the native structure ensemble to those of the mutated structure ensemble, and begin to quantify the effects of different mutations on protein structure. For example, p_aa_pp and two of the gdtmm terms are important for correct classification, while the impact of the ref term is negligible (see Results for further details).

### Training Data Generation

The score terms and score term distributions derived from the relax runs, while useful in themselves, undergo two transformations before they can be used in the machine learning algorithm that ultimately predicts which mutations will result in a ts phenotype. We applied a metric for comparing wild-type and mutant score term distributions, and we converted these measurements into features that are used to train our classifier. We also added several non-Rosetta features to our training data. These steps are described below.

#### Comparing quartiles of score distributions

**Figure 5:**
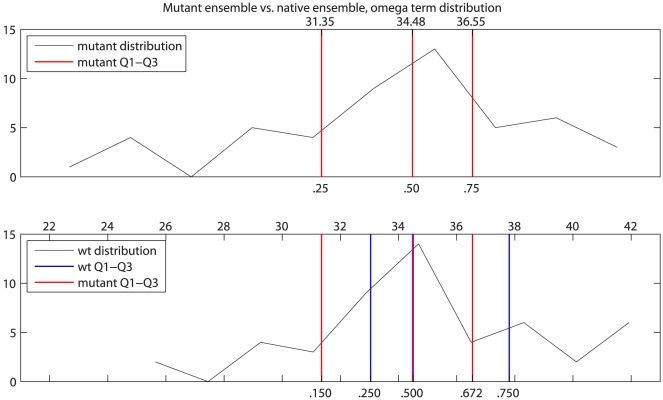
Quartile method for comparing distributions of Rosetta score terms. Mutant ensemble quartiles 1–3 were calculated for the mutant ensemble distribution (top) of the omega score term, which measures deviation of the 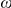 bond angle from its ideal of 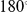. Q1–Q3 are indicated by red lines, with the corresponding values above and percentiles below. The mutant Q1–Q3 values were then mapped to locations in the wild type (wt) ensemble distribution (bottom). Q1–Q3 of the mutant distribution are again indicated by red lines, with their percentiles relative to the wt distribution shown below. Wild type ensemble Q1–Q3 are shown in blue for reference.

Comparing native and mutant distributions of terms derived from Rosetta relax runs in a way that is applicable across all proteins is challenging for reasons mentioned earlier: the tremendous range of protein structure, function, and starting energies, and the protein-dependent change in 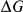 that may result in a ts phenotype. To correct for these differences we employ a quartile-based method of comparing mutant relax score ensembles to native relax score ensembles, effectively normalizing for differences in starting structure energy terms. We chose a quartile-based approach that allows us to compare distributions without making assumptions about the underlying distribution of the data (Fig. 5). The procedure calculates quartiles 1–3 in the mutant ensemble, then expresses those values as percentiles within the native ensemble. Specifically, for a given mutation ensemble E and set of values S for a single score component of E, we found the first, second, and third quartiles (Q1, Q2, and Q3) of S (that is, we sorted the S values from low to high, then found the values at positions 0.25*| S|, 0.5*| S|, and 0.75*| S|). We then found the percentiles of those Q1, Q2, and Q3 values within the set of values S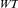 of the same score term in the wt ensemble. Percentiles were represented as fractions between 0 and 1, and values lying outside those ranges were clamped to 0 or 1 as appropriate.

As an example, the analysis shown in Fig. 5 of the omega score term, which measures the deviation of the 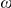 bond angle from its ideal of 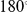 (see Table 1) proceeds as follows. The omega score term values from the mutant ensemble are analyzed, and found to have values (Q1, Q2, Q3) = (31.35, 34.48, 36.55). These three values are then located within the wt ensemble, and each is given a number corresponding to its percentile with respect to the omega term distribution in the wt ensemble. The result is a set of feature values (omegaQ1, omegaQ2, omegaQ3) = (0.150, 0.499, 0.672), so that the omega scores from the individual runs in the ensemble are now represented by these three percentile values. These values indicate that the means of the mutant and native distributions are the same but the first and third quartiles of the mutant are shifted downward relative to native. Applying the quartile method yielded three input features for each Rosetta score term in the mutant ensemble, for a total of 93 features from 31 score terms.

#### Additional features used in predicting ts mutations

The final score file contains three types of features: Rosetta score-based features (Table 1), additional structure-based features (Table 2), and sequence-based features (Table 3). The Rosetta score term-based features were described above. The additional structure-based features include the raw ACC value (percent side chain accessible, feature ACCP) and three features denoting whether the native residue participates in an 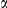-helix, a 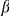-sheet, or a loop region (features ss_H, ss_S, and ss_L).

**Table 2:** Non-Rosetta structure-based features.

Feature	Description
ACCP	solvent-accessible surface area (ACC)
ss_H^3^	secondary structure: 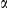 -helix
ss_S^3^	secondary structure: 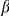 -sheet
ss_L^3^	secondary structure: loop region

3 Worsened performance.

Structure-based features not based on Rosetta score terms. Superscripts denote features removed from the final training set.

**Table 3:** Sequence-based features.

aminochange^1^	four-category change in amino acid: 0 = same amino acid, 1 = different amino acid in same category, 2 = different category
aminochange^2^	seven-category change in amino acid
pssm_mut	log-likelihood of mutated amino acid from position-sensitive scoring matrix
pssm_nat	log-likelihood of native amino acid from position-sensitive scoring matrix
pssm_diff	difference in log-likelihood of mutated and native amino acid
freq_mut^1^	frequency of mutated amino acid in multiple sequence alignment
freq_nat^1^	frequency of native amino acid in multiple sequence alignment
freq_diff^1^	difference in frequency of mutated and native amino acid
info_cont	position information content from PSI-BLAST

1 Removed due to high correlation with other feature(s).

2 Worsened performance.

Sequence-based features from BLAST, PSI-BLAST, or other analysis. Superscripts denote features removed from the final training set.

Sequence-based features were derived using only amino acid sequences. We first created two categorizations of amino acids into groups (large hydrophobic, polar, charged, etc.), one with four categories and another with seven (features aminochange, aminochange2). From these we derived two features, one for each group, denoting whether the amino acid at the site in question remained the same (no mutation; native), was mutated to a residue in the same category (e.g., one polar residue to another), or changed categories completely (e.g., polar to charged or small hydrophobic to large hydrophobic). Finally, we calculated a set of features using BLAST [17] and PSI-BLAST [18]. For each protein, we ran one iteration of BLAST on the NCBI non-redundant protein sequences (nr) database [19], then performed one iteration of PSI-BLAST to generate the position-specific scoring matrix (PSSM) for the protein. From the PSSM and other PSI-BLAST statistics, we derived seven features based on information content and native and mutated residue log odds and frequencies (features info_cont, pssm_mut, pssm_nat, pssm_diff, freq_mut, freq_nat, freq_diff).

The resulting training file contains 109 features: the class label (ts or non-ts, not used as a predictor or feature) and the 108 numeric features described above. We found significant correlation among some of the features in this set, and pruned them to a minimal informative set as described below.

### Training and Validation

The data generation steps above produce an input file with one line per run ensemble, where each line has values for each of the Rosetta score term-based, additional structure-based, and sequence-based features described above (conceptually this is equivalent to a matrix where each row represents a run ensemble and each column represents one input feature). This file provides the input for training our machine learning algorithms.

We used the Weka [20] suite of machine learning tools and the libSVM package for our classifier training and testing. Weka is a Java-based program that provides command-line and GUI access to multiple data formats, supervised and unsupervised classifiers, filters, evaulation metrics, and pre- and post-processing tools. libSVM is a support vector machine library that provides access to different SVM implementations in several programming languages and can be used directly from programs such as Weka and Matlab. Using Weka and libSVM, we evaluated several different types of classifiers, concentrating on SVMs and variations of the C4.5 [21] tree-based classifier before choosing the support vector machine as the most accurate and stable for this task.

Support vector machines describe a family of methods for performing statistical inference from data, generally regression or classification [22], [23]. A two-class SVM classifier assigns labels based on the sign of the decision function
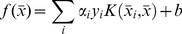
(1)where 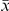 is the sample to be classified, 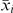 are the training samples, 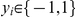 are the class labels for each 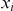, 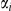 are weights assigned to each sample during training, 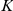 is the kernel function, and 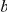 is a bias term. For a linear SVM, the kernel function is the dot product 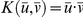, and the decision function can be written as
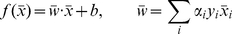
(2)If the training data are 2-dimensional vectors (*i.e.*, points in the plane), 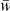 and 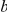 are the normal vector and intercept describing the line that separates the positive and negative training samples with the largest possible margin between the two classes (a maximum margin hyperplane). 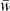 is formed by a weighted sum of the training samples; since most of the 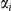 are set to zero during training, this sum is over a small subset of samples referred to as the support vectors of the classifier. Data that are not linearly separable in the input space are accommodated in two ways: First, per-sample “slack” terms are introduced that penalize samples that fall on the wrong side of the decision boundary; a user-specified “complexity” parameter 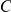 controls the trade-off between penalizing incorrect classifications and maximizing the margin between classes. Second, kernel functions other than the linear kernel can be used to map the training samples into a higher-dimensional space in which they may be linearly separable. An explicit mapping 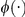 corresponds to a kernel function of the form 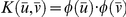. However, kernels that satisfy Mercer's condition can compute this quantity directly from vectors 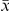 and 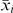 in the original input space without explicitly computing the transform 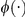, allowing efficient calculation of mappings to higher-dimensional (or even infinite-dimensional) spaces. Common kernels are the homogeneous polynomial kernel 
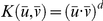
 and the radial basis function (RBF) kernel 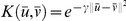. In this work, we employ linear and RBF kernels.

#### Classifier Selection and Evaluation

We tested two types of support vector machine: SVM-LIN, a straightforward SVM using a linear kernel, and SVM-RBF, which uses a (non-linear) radial basis function kernel. Here we describe results from SVM-LIN, SVM-RBF, and an SVM-RBF variant called SVM-seq. SVM-LIN is simpler, using a linear kernel in the original input space, and allows straightforward determination from a trained classifier of the features most important for classification. SVM-RBF uses an RBF kernel that results in non-linear decision boundaries that more exactly find the intermediate ts range between wt and lethal mutation; this makes it more accurate but less robust to small changes in the training set. SVM-seq uses the same type of radial basis function as SVM-RBF, but is given a distinct name because it uses a subset of the full input set that excludes all structure-based features. The testing of both linear and non-linear classifiers here is intended to provide a mix of complexity, interpretability, and accuracy.

We used a variant of cross-validation (CV) to evaluate the accuracy of our SVM-LIN, SMV-RBF, and SVM-seq classifiers. Cross-validation allows the use of a single set of samples as both training and test set by making multiple partitions of the starting set into training and testing sets. For example, 10-fold CV makes 10 splits of the starting set into 90% training, 10% testing, in such a way that the ten 10% splits are disjoint and their union is the entire data set. It then trains and tests on each 90/10 split in turn and reports the aggregate statistics over all 10 splits. In this way it uses the entire starting set for training and testing without ever testing on a sample that was also used for training. Performing 10-fold CV 10 times (10×10-fold CV) with different random seeds is more robust: since the overall set of samples is partitioned into different sets of 90/10 splits each time, the results will vary allowing the calculation of a mean and variance on the aggregate statistics. Finding optimal SVM hyper-parameter values (see below) requires a more stringent procedure: we make five 80/20 splits of the starting set, ensuring again that the 20% splits are disjoint and cover the entire data set. We then set each 20% split aside as a “leave-out” set, perform 10-fold 10× CV on the 80% to evaluate hyper-parameter values, then report results on the leave-out set. This ensures that accuracy is never calculated on samples that were used for finding hyper-parameters. We refer to this as “5× leave-out CV”.

#### Parameter and Feature Selection

Given the above means of training and evaluating our classifiers, we took several steps to improve their performance. Both SVM implementations have hyper-parameters that can be tuned to achieve optimal predictive accuracy. SVM-LIN and SVM-RBF have a complexity parameter 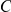 that specifies the penalty for non-separable samples. Higher values of 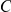 cause the training process to better fit the training samples: for linear SVM-LIN, this increases the weight on the samples closest to the decision boundary between the classes; for SVM-RBF, this results in a more complex decision boundary between ts and non-ts classes. 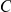 must be high enough to distinguish between the two classes, but not so high that the classifier learns the noise and outliers in the training set and therefore does not generalize well to novel samples. SVM-RBF also has a 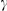 parameter that affects the area of influence of the radial basis functions. As with the complexity parameter, higher values lead to more convoluted decision boundaries by making basis function influence more local.

**Figure 6:**
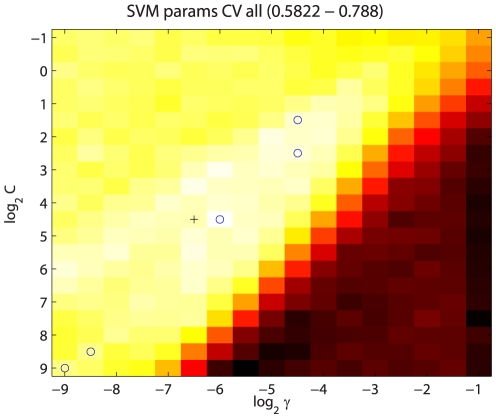
SVM-RBF parameter space. SVM-RBF precision on the ts class is shown as a function of 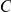 and 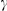 parameters. Values shown are the mean across the five leave-out CV runs, and range from 0.5822 to 0.788. Blue circles indicate the parameter values yielding the highest ts precision for each of the five leave-out CV runs. The final median 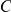 and 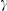 values are indicated by the black cross. While the optimum parameter values across the five leave-out CV runs differ, they are all located along the “valley” of high precision that is visible running from upper right to lower left, indicating that multiple combinations of 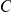 and 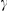 values lead to classifiers having similarly good performance.

We performed a simple search in parameter space, using 5× leave-out CV to evaluate different hyper-parameter values at set intervals in log-transformed parameter space (*e.g.*, 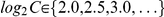). This gave a distribution of hyper-parameter values across the leave-out CV sets. What we found was that the choice of samples in the training and testing sets made much more of a difference than the parameter values, and that there was generally a wide range of parameter values with roughly equivalent performance (Fig. 6). Our final parameter values were the median values across the five leave-out CV sets. This same method also yielded our final precision figures as described in Results.

We removed features that were either confounding or redundant from the training set to improve both performance and interpretablity. We examined all strongly correlated (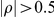) feature pairs, and tested the effect of removing each of these features one at a time with five variants of our SVM classifiers using 10×10-fold CV. Features derived from a common Rosetta score term (*e.g.*, omegaQ1, omegaQ2, and omegaQ3) were included or removed as a group. We identified three sets of redundant Rosetta-derived features, listed by Rosetta term: { fa_dun, fa_atr, fa_sol, fa_intra_rep }, { gdtmm7_4, maxsub }, and { gdtmm2_2, rms }. After testing as described above, we kept the features from the first term listed in each group and removed the rest. We also identified four pairs of correlated sequence-based features: { aminochange2, aminochange }, { pssm_mut, freq_mut },{ pssm_nat, freq_nat }, { pssm_diff, freq_diff }. We then tested all remaining non-Rosetta derived features to evaluate how their inclusion affected performance using the same procedure as above. We found that the following features actually degraded performance, and removed them: ss_H, ss_S, and ss_L. Finally, we removed the irms set of features, as irms term values were always zero. In all, 26 features were removed, giving a final training set consisting of 86 features.

### Detailed Protocol

The following step-by-step instructions will reproduce our ts prediction protocol on any computer with a UNIX command line. These instructions follow the protocol capture, which is available as part of the Rosetta 3.3 protein modeling suite available at http://www.rosettacommons.org. The following must be installed: Rosetta release 3.0, Weka 3.6 or better, libSVM 2.8.9 or better, Probe 2.12 or better, Python 2.6, PyMOL 1.2 or better, NCBI BLAST+ tools 2.2.22 or better with the “nr” database, sed, and awk. We also assume there is a starting model in Protein Databank format. In the following example, the protein is YBR109C (yeast calmodulin), and the file is YBR109C.pdb, a homology model based on 1LKJ (NMR structure of yeast apo calmodulin). All commands should be executed in the top-level directory of the protocol capture archive. All scripts referenced reside in the scripts/subdirectory of the capture.

The ts prediction protocol is split into three stages: generating scripts for the Rosetta runs, performing the Rosetta runs, and making predictions based on the run output. This separation is made because these runs are typically performed on clusters: commands for submitting jobs to clusters vary depending on the cluster software and configuration, and runs may be submitted from a different machine than that used for the generation and prediction stages. We will address each of these three stages in turn.

Scripts are generated using the generate-scripts.sh command as shown below. The -protein argument specifies the name of the protein, and must be the same as the name of the starting structure file without the .pdb extension. -species is used in larger ts prediction runs that involve Rosetta runs over many proteins, such as cross-validation on the training set, and will show up in the prediction results. -cutoff gives the threshold for selecting positions to mutate in the starting structure (typically this is 10, but we use 0 to reduce the number of run scripts generated in this example). mini_bin and mini_db give the full paths of the Rosetta binary and database directories, respectively, on the machine where the Rosetta runs will be executed.

scripts/generate-scripts.sh -protein YBR109C -species Scer -cutoff 0 -mini_bin

∼/rosetta-3.0/bin -mini_db /rosetta-3.0/rosetta3_database

The output of generate-scripts.sh is a collection of scripts to perform Rosetta relax runs: one script for the native structure, and one for each mutation at each position. The command above, with an ACC cutoff of 0, finds four sites meeting the ACC threshold, and generates 77 scripts: one for the native structure and one for each of the 19 mutations at each of the four sites. The native structure script will be called YBR109C-WT.sh, and the mutatued structure scripts follow the convention YBR109C-aNNNb.sh, where a is the native residue identity, NNN is the residue position, and b is the mutated residue identity. When executed, these scripts will perform the relax runs and create the score files required to generate the input files for classification. These runs will typically be performed on a cluster. Commands for submitting jobs on clusters vary depending on the cluster management software installed; the following command line is appropriate submitting from a Bash shell to a cluster running TORQUE:

for a in *.sh; do qsub -d $(pwd) $a; done

Each run calls two different Rosetta protocols, relax and score. Sample command lines for the mutation F140A (mutating PHE at position 140 to ALA) are shown below. The first command generates an ensemble of 50 relax runs, and the second re-scores the structures to generate additional score terms (such as the gdtmm series of terms):

∼/rosetta-3.0/bin/relax.linuxgccrelease -database /rosetta-3.0/rosetta3_database

-s YBR109C-F140A.pdb -native YBR109C.pdb -nstruct 50 -relax:fast

-out:file:scorefile YBR109C-F140A.sc -out:pdb_gz

∼/rosetta-3.0/bin/score.linuxgccrelease -database /rosetta-3.0/rosetta3_database

-s YBR109C-F140A_????.pdb.gz -in:file:native YBR109C.pdb -in:file:fullatom

-out:file:scorefile YBR109C-F140Arescore.sc

The rosetta3_database directory is part of the Rosetta 3.0 install. The relax run input file YBR109C-F140A.pdb is generated along with the Rosetta run scripts, and follows the same naming convention for mutated residues. The relax run output file YBR109C-F140A.sc and the score run output file YBR109C-F140Arescore.sc are the inputs for the final prediction stage.

Once the runs are complete, predict-ts.sh is used to analyze the output and predict which mutations will have a temperature-sensitive phenotype. This script merges information from the relax and score run output files, produces an input file for the classifiers, and performs classification on the input samples. Again, the -protein argument is used to specify the protein name:

scripts/predict-ts.sh -protein YBR109C

The output of the prediction step will be two text files: for this example, these files are named YBR109C-svmlin.txt and YBR109C-svmrbf.txt. These files show the predictions made by SVM-LIN and SVM-RBF, respectively, with each line giving the absolute rank, confidence, and mutation. The mutation field shows the protein name, the native residue identity (1-character code) and position, the mutation made (3-character code), and the species abbreviation. Below are the top five predictions made by SVM-LIN on YBR109C:

rank conf id

1  0.839 YBR109C-F140_GLY_Scer

2  0.831 YBR109C-F140_ASP_Scer

3  0.783 YBR109C-F140_CYS_Scer

4  0.776 YBR109C-F140_PRO_Scer

5  0.769 YBR109C-F140_THR_Scer

## Results

Our primary means of evaluating our method was examining the ts predictions from our 5× leave-out CV runs. We evaluated all methods according to four metrics: precision, significance, correlation, and area under the ROC curve (AUROC).

Our 5× leave-out CV method, in addition to finding optimal SVM hyper-parameters, yielded a conservative estimate for ts prediction precision. By tallying the number of correct and incorrect predictions on each leave-out set, we calculated precision across the entire set. Precision for both classifiers is significantly better than random, with SVM-RBF slightly outperforming SVM-LIN (precision of 0.795 and 0.745, respectively).

We also looked at per-species and multi-species effects. Breaking down the above results by species showed lower precision for *C. elegans*, where we only had 5 ts samples, and perfect prediction for *D. melanogaster*, which entirely lacks non-ts samples. Accuracy for both *C. elegans* and *S. cerevisiae* was significantly lower when training only on that species' samples as opposed to the full multi-species training set, with *C. elegans* precision dropping to nearly zero using single-species training. While some of the improvement from multi-species training is certainly due to the increase in training set size in the multi-species setting, the marked improvement in *C. elegans* performance strongly suggests that our technique is also extracting species-independent rules. Mixed-species training appears to significantly increase the range of proteins for which we can make accurate predictions.

To further quantify our method's performance, we developed a heuristic to estimate p-values for each method and species. Again, *C. elegans* had the weakest results, due again to the small number of ts samples; and fly predictions alone were not significant as there were no non-ts samples. However, yeast prediction was significantly better than random, and overall prediction was at 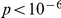.

**Figure 7:**
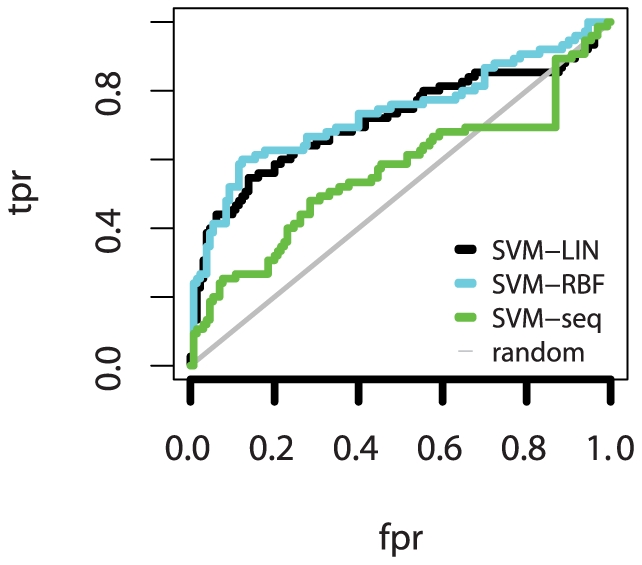
Classifier Performance. The Receiver-operating characteristic (ROC) curve is shown for SVM-LIN, SVM-RBF, and SVM-seq (RBF classifier trained only on sequence data). ROC curves for each classifier showing false positive rate (fpr) and true positive rate (tpr), with the reference line for random classification is shown in gray. The difference between each classifier and the reference line shows the improvement over random of our method. The steep slope at the lower left of the classifier curves indicates that the highest-ranked predictions are most likely to be accurate for all three classifiers. Area under curve: SVM-LIN = 0.713, SVM-RBF = 0.734, SVM-seq = 0.563.

An additional measure of our improvement over random is given by classifier performance curves: the receiver operating characteristic (ROC) curve and the precision-recall (PR) curve, as well as the area under each (AUC and AUPR, respectively) (Fig. 7). The ROC curve is a standard method in machine learning to visualize the performance of a classifier that relies on the confidence or estimated probability that both SVM-LIN and SVM-RBF assign to each prediction. PR curves are similar to ROC curves, but impose a higher penalty for highly-ranked incorrect predictions. The ROC curve shows the relationship between the rate of correct predictions and the rate of incorrect predictions, and the area under the curve is equivalent to the probability that a randomly chosen ts sample will be ranked higher that a randomly chosen non-ts sample. The steep portion at the lower left of the ROC curve shows that our top-ranked predictions are particularly accurate, which is ideal since our goal is to use the top few (generally 5) predictions in our ranked list. While SVM-seq performance in the top few predictions is similar to that of SVM-LIN and SVM-RBF, SVM-seq performance degrades rapidly for both ROC and PR curves, as reflected by the areas under the curves.

We also analyzed the accuracy of our rankings by using the point-biserial correlation to calculate how well the classifier confidence scores correlated with actual correctness of prediction. If 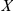 is a vector representing the correctness of each prediction (0 = incorrect, 1 = correct), 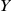 is the corresponding equal-length vector of confidences (predicted probability of ts) for each prediction, and 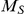 is the mean value of 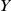 for incorrect (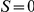) and correct (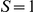) predictions, then the point-biserial correlation is defined as 
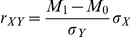
. Both methods show reasonable correlation, indicating that correct predictions are more likely to be ranked higher, increasing the likelihood of finding correct predictions at the top of the list. SVM-RBF, despite having higher precision, has lower correlation, which may indicate some over-fitting.

We next examined the highest-weighted features of SVM-LIN – in other words, those that were most important in determining the label assigned to each sample. For each of the five cross-validation leave-out sets, we examined the weights assigned to the input features, and examined the top features ranked by absolute weight value. Top features varied somewhat across the CV leave-out sets; averaging rankings of features across leave-out sets gave to following ordered list of top five features: aminochange2, Repack_stdev_scoreQ2, gdtmm4_3Q2, p_aa_ppQ3, and gdtmm7_4 (see Tables 1 and 3). The 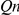 at the end of the feature name denotes the feature derived from the 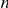
th quartile comparison, except for aminochange2 which is not derived from a Rosetta score term and gdtmm7_4 where Q2 and Q3 appeared alternately. Feature aminochange2, which abstracts the actual change in amino acid to a change in amino acid class (*e.g.*, hydrophobic to polar; see Table 3), was first in all splits. Repack_stdev_scoreQ2 is always in the top five: the Rosetta term Repack_stdev_score tracks how much a structure varies throughout the relax run, and Q2 denotes the second quartile comparison feature generated from that term. p_aa_ppQ3, which gives the probability of an amino acid given its observed phi and psi angles, appeared the top five in four splits, and the gdtmm7_4Q2 and gdtmm7_4Q3 features, which track overall movement of protein atoms from the positions in the starting structure, appeared the top five in multiple splits. The signs of the weights are the same in all cases, meaning that each feature listed above always favors the same label (ts or non-ts). We can use these top-ranked features and their weights to describe the area of intermediate destabilization between neutral and loss-of-function mutations that is implicit in SVM-LIN: changes in amino acid class are strongly favored, and some movement of atoms from their native position and an increase in overall energy are also favored. However, structures that do not settle on a stable conformation during the relax process and structures with unlikely local structure (p_aa_pp) are strongly disfavored, and structures with significant movement of atoms from native are less strongly unfavorable.

Previous predictive methods have predicted ts mutations from sequence alone. Since we added structure-based features at some computational expense, we wanted to quantify the improvement achieved by adding these features. We compared the performance of our SVM-LIN and SVM-RBF methods to a method we call SVM-seq, which is a variant of SVM-RBF that has been trained (including tuning of hyper-parameters) without any structure-based features. While precision and correlation compare favorably, the SVM-seq predictions lack significance. This is caused by a high false negative rate: many ts samples are incorrectly labeled non-ts, and few samples are predicted to be ts (roughly 20% of the number predicted as ts by SVM-LIN and SVM-RBF). In addition to the reduced significance caused by the small number of ts predictions, SVM-seq is also considerably less stable in the predictions that it makes: precision varies considerably among the five CV leave-out sets, from 0% to 100%, with one leave-out set producing no ts predictions at all. This poor behavior on the test sets across the CV leave-out sets may reflect consistent over-fitting of the training data by SVM-seq.

## Discussion

We have developed and tested a computational method for predicting temperature-sensitive mutations from protein structure, presenting results using the two support vector machine classifiers SVM-LIN and SVM-RBF. While both classifiers perform well, SVM-LIN may be better for the particular task of predicting a very small number of highly accurate mutations. This opinion is based primarily on examination of the point-biserial correlations: while SVM-RBF is slightly more accurate overall, its lower point-biserial correlation suggests possible over-fitting of the training data. Though SVM-RBF performs better than SVM-LIN at the very top of the prediction list, both are strong, and SVM-LIN is more likely to generalize in a stable fashion to new proteins or organisms. Overall, this suggests that SVM-LIN is more appropriate for the “top 5” predictive task described here, though SVM-RBF may have a higher yield when making a larger number of predictions. The interpretability of SVM-LIN in terms of features important for classification is also quite attractive. SVM-RBF may become superior in the long run as training data become more plentiful and reliable.

As described in Methods, our pipeline uses the Rosetta “fast relax” protocol for structure prediction after amino acid substitution. This protocol can be slow, as it involves Monte Carlo search of the entire space of backbone and side chain angles. A recent investigation of the correlation of experimentally determined and Rosetta-generated 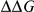 values after single-residue substitutions [9] tested 20 different combinations of strategies for searching conformational space and resolution of the Rosetta energy function. Each of these protocols was assessed using its correlation to the experimentally determined values and the effect of the mutation (stabilizing, neutral, or destabilizing). Many strategies that are considerably less time-consuming than the full Monte Carlo ensemble generation had equally good correlation, such as simple all-residue repacking with a soft repulsive van der Waals term. In the near future we plan to evaluate some of these protocols in place of the current “fast relax” protocol in our method to see if equivalent results can be generated using significantly less computing time.

We also plan to make our ts prediction pipeline available as a public web resource. Our ts prediction resource will make it possible for users to upload the structure of a protein of interest (obtained experimentally or via homology modeling) and choose several evaluation options such as solvent-accessible surface area cutoff. Prediction jobs will be sent to a small cluster for asynchronous evaluation. On completion of a job, the user will be notified, and results will be made available in the form of a ranked list of ts predictions. Our hope is that the availability of such a resource will contribute significantly to the discovery of ts mutations, as well as adding size and stability to our ts prediction training set.
